# Case Report: A Novel ARMC5 Germline Mutation in a Patient with Primary Bilateral Macronodular Adrenal Hyperplasia and Hypogammaglobulinemia

**DOI:** 10.3389/fgene.2022.834067

**Published:** 2022-03-15

**Authors:** Walter Vena, Valentina Morelli, Maria Carrabba, Francesca Elli, Giovanna Fabio, Ilaria Muller, Camilla Lucca, Maria Antonia Maffini, Andrea Gerardo Lania, Giovanna Mantovani, Maura Arosio

**Affiliations:** ^1^ Endocrinology Unit, Fondazione IRCCS Ca’ Granda Ospedale Maggiore Policlinico, Milan, Italy; ^2^ Unit of Endocrinology, Diabetology and Medical Andrology, IRCCS, Humanitas Clinical Research Hospital;Milan, Italy; ^3^ Department of Internal Medicine, Rare Diseases Unit, Primary Immunodeficiencies Centre for Adults, Foundation IRCCS Ca’ Granda Ospedale Maggiore Policlinico, Milan, Italy; ^4^ Department of Clinical Sciences and Community Health University of Milan, Milan, Italy

**Keywords:** adrenal hyperplasia, ARMC5, hypercortisolemia, hypogammaglobulinemia, infections

## Abstract

Primary bilateral macronodular adrenal hyperplasia (PBMAH) represents an uncommon cause of endogenous hypercortisolism. Since the first description in 2003 in a French cohort, many papers have been published describing families as well as isolated individuals affected with this condition, who were found to harbor a genetic variants in the armadillo-repeat containing 5 (*ARMC5*) gene, a tumor-suppressor gene with a still unknown role in the disease pathogenesis. Studies in rat models suggested a possible link between *ARMC5* damaging variants and the impairment of the cell-mediated immune response, leading to a higher susceptibility to bacterial and viral infections. To our knowledge, we describe the first case of a patient affected by PBMAH with hypogammaglobulinemia and monthly relapsing human herpes simplex viral infections. After the detection of subclinical Cushing’s syndrome, a unilateral laparoscopic adrenalectomy was performed. Subsequent genetic analysis of *ARMC5* performed on genomic DNA extracted both from the adrenal tissue and lymphocytes revealed a novel somatic frameshift variant in exon 1 (c.231_265del:p.A77Afs*13) and a novel germline variant in exon 6 (c.2436del: p. C813Vfs*104). After adrenalectomy, we observed a significant improvement of clinical features concerning both hypercortisolism and relapsing viral infections, thus suggesting a possible adjuvant role of hypercortisolism on a genetic-based derangement of the immune system.

## Introduction

Primary bilateral macronodular adrenal hyperplasia (PBMAH) is an uncommon cause of endogenous glucocorticoid (GC) excess, that can either be responsible of asymptomatic forms of subclinical hypercortisolism (SH) or lead to an overt adrenocorticotroph hormone (ACTH)-independent Cushing’s syndrome (CS) (De Venanzi et al., 2014). Since the first identification and description by Assie and colleagues (Assié et al., 2013) in 2013 in a cohort of PBMAH patients, the tumor suppressor gene armadillo-repeat containing 5 (*ARMC5*) raised growing interest in the scientific community worldwide. *ARMC5* deleterious variants were described as associated with PBMAH with a prevalence ranging from 10 to 55% in different case series and it was hypothesized to be a key factor in the development of more severe forms (Fragoso et al., 2015; Stratakis and Berthon, 2019), possibly requiring a more aggressive clinical approach. The exact role of *ARMC5*-encoded protein in PBMAH pathogenesis is still under investigation, but *in vitro* studies proved that homozygous and/or compound heterozygous inactivating variants could be responsible for an altered apoptosis in adrenal cells, an aberrant expression of some receptors (e.g. of LH, vasopressin), along with a focal ectopic intra-nodular production of ACTH (Louiset et al., 2013). Preclinical studies recently demonstrated a widely distributed expression of this protein in adult human tissues, suggesting a possible role in the development of tumors other than PBMAH, such as meningiomas (Elbelt et al., 2015). The *ARMC5* knock-out (KO) mouse model showed bilateral adrenal enlargement, increased glucocorticoid levels, similarly to what observed in PBMAH patients, and a significant impairment of the T-cell function (Hu et al., 2017). Glucocorticoids are key regulators of the immune system activation by multiple mechanisms affecting both humoral and cell-mediated responses, but the knowledge of the effect of endogenous GCs on immunoglobulins (Igs) concentration is scarce and limited to specific clinical contexts (Cain and Cidlowski, 2017). Here we describe, for the first time, a case of concomitant presence of T-cell function impairment and SH in a patient with PBMAH carrying both germline and somatic novel variants of the *ARMC5* gene.

**FIGURE 1:**
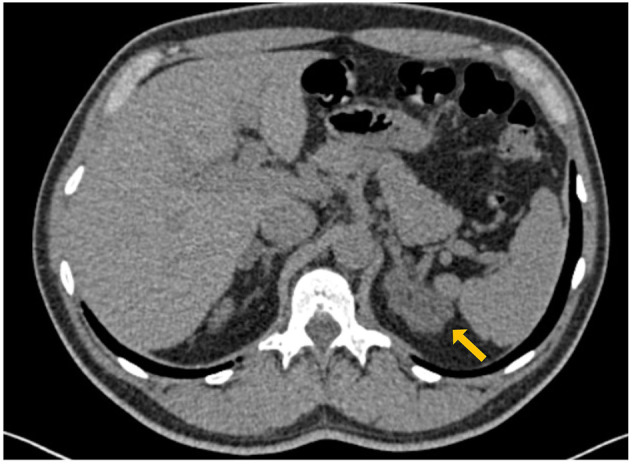
Abdomen CT scan showing bilateral enlargement of adrenal glands with a typical macro-nodular aspect of left adrenal (maximum diameters were 45 and 16 mm at left and right side with low Hunsfield Unit density (HU −20/+12), respectively).

A 56 years old man was referred to our Endocrinology Unit for the incidental finding of a bilateral adrenal enlargement discovered during computed tomography (CT) scan investigation during the workup of a recently diagnosed slight hypogammaglobulinemia (Figure 1). Patient’s medical history revealed monthly recurrent labial herpes simplex virus (HSV) infections since adolescence, a recent episode of thoracic shingles, a previous Helicobacter Pylori-associated gastritis, and the recent onset of high blood pressure, controlled by low dose β-blocker.

## Investigations

Because of monthly occurrence of labial HSV, the patient underwent immunological laboratory evaluation, that showed a normal neutrophils count, normal serum IgA levels, slightly low serum IgG and IgM levels. The T- and B-cells immunophenotype found a moderate increase in CD3+ T-cells, with normal CD4+ and CD8+ subsets and a normal count of NK and CD19+ B cells (Table 1). Abdomen magnetic resonance imaging (MRI) evaluation confirmed the involvement of both left and right adrenal glands with a lipid-rich multi-nodular hyperplasia, enlarged to 45 and 16 mm (maximum diameter) respectively. Physical examination did not reveal any specific feature of CS (buffalo hump, moon face, easy bruising purple or red striae) or abnormal anthropometric measures (body mass index 25.6 kg/m^2^, waist circumference 92 cm). A complete laboratory workup raised the suspect of subclinical ACTH-independent hypercortisolism as serum cortisol suppression failed both after 1 mg dexamethasone-suppression-test (1 mg DST; 13.4 ug/dl, expected value < 1.8 ug/dl) and low dose Liddle suppression test (LDDST; 10.4 ug/dl), in the presence of basal undetectable ACTH levels (<5 pg/ml), with urinary free cortisol levels within normal ranges (<100 ug/24 h) on several determinations. Other basal parameters of adrenal function excluded primary aldosteronism (serum aldosterone 13.4 ng/dl, plasma renin 8.0 U/mL, K 4.4 mmol/L) and plasma 17-hydroxyprogesterone response after intravenous injection of 250 mg Synacthen test ruled out the presence of 21-hydroxylase deficiency. Moreover, other androgens levels were within normal ranges.

**TABLE 1:** Patient’s immunological laboratory evaluation before and after surgery.

	Baseline	Post-surgical control
**Leucocytes 10e3/mmc**	5.58	6.57
**Lymphocytes 10e3/mmc**	2.45	3.03
*CD3 + TCell/µl (700–2,100)*	***2,421***	***2,669***
*CD4*+ *TCell/ul (300–1,400)*	1,344	1,338
*CD8*+ *T Cell/ul (200–900)*	773	*815*
*CD19*+ *B Cell/µl (100–500)*	268	264
*CD16*+*CD56*+*CD3-NK Cells/µl (90–600)*	234	240
**IgA levels mg/dl (70–400)**	104	115
**IgG levels mg/dl (700–1,600)**	**616**	745
**IgM levels mg/dl (40–230)**	**32**	**33**
*IgG1 g/L (3.150–8.500)*	*3.233*	*3.405*
*IgG2 g/L (0.640–4.950)*	*2.132*	*2.977*
*IgG3 g/L (0.230–1.096)*	*0.428*	*0.610*
*IgG4 g/L (0.110–1.570)*	*0.382*	*0.509*

## Treatment and Genetic Investigations

**FIGURE 2:**
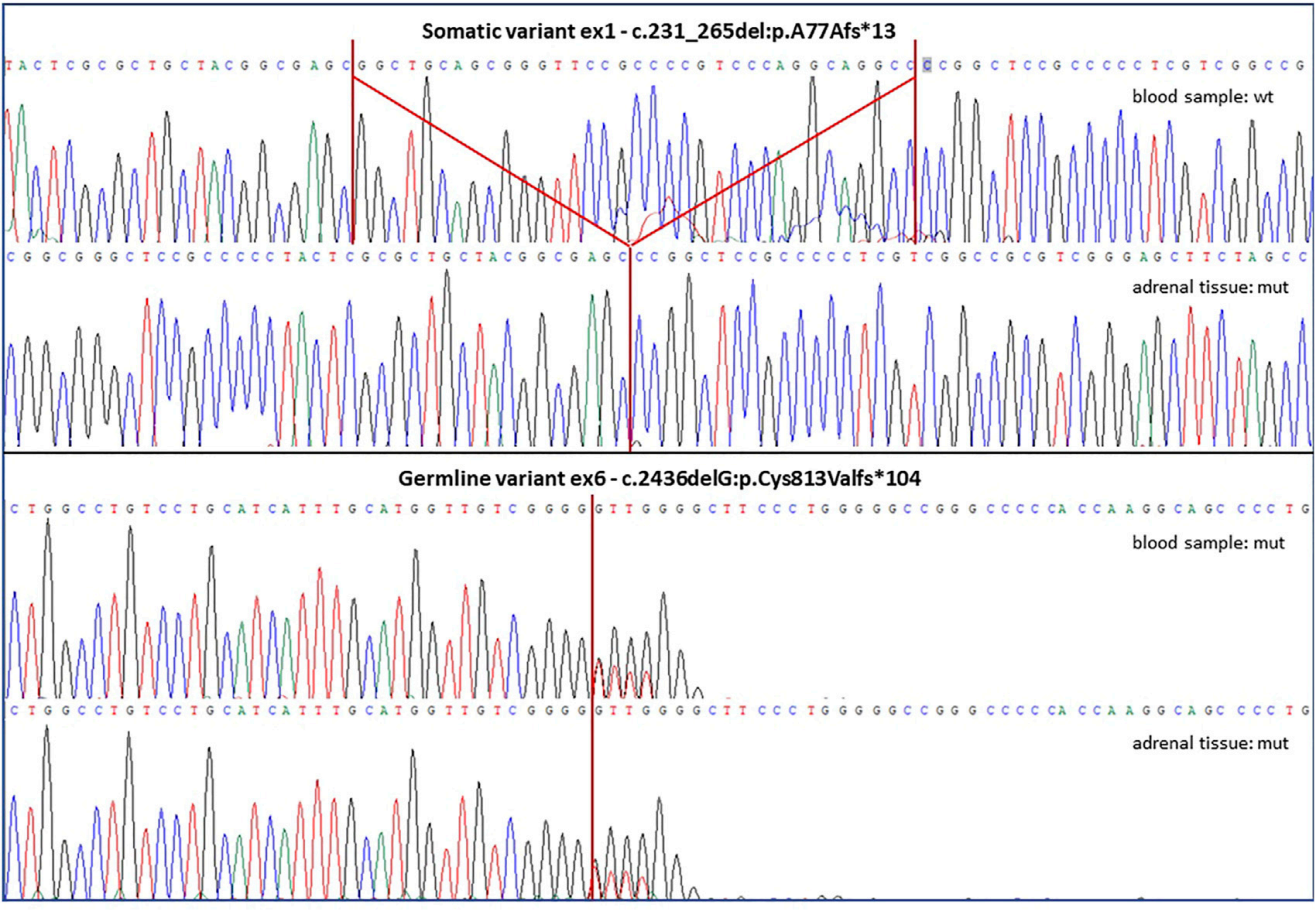
The figure shows altered *ARMC5* sequences discovered in both lymphocyte-derived and adrenal tissue-derived gDNA samples of the described patient. The somatic variant was found only in the tumor sample and it was a deletion of 35 bps in exon 1, while the germline variant was detected in both blood and tumor samples and it was a single base deletion in exon 6. Both genetic variants are novel to the literature and predicted as highly damaging and pathogenetic, as both were frameshift mutations causing the generation of and altered protein sequences and premature stop codons.

The patient was addressed to surgical treatment consisting in laparoscopic left adrenalectomy. The histopathological examination was consistent with the diagnosis of a macronodular adrenal hyperplasia, thus prompting genetic testing of the *ARMC5* gene. Genomic DNA (gDNA) extracted from peripheral blood leucocytes was analyzed by Sanger sequencing and we identified a novel heterozygous genetic variant (c.2436del; p. C813Vfs*104) in exon 6 (all primers and thermal conditions available upon request). The examination of tumor-extracted gDNA revealed both the presence of the germline variant in exon 6 and of an additional c.231_265del -p.A77Afs*13 homozygous variant in exon 1 (Figure 2). Both molecular alterations determined a frameshift, generating premature stop codons and protein truncations. They were classified as pathogenetic variants after *in silico* prediction studies by The Human Genomic Variant Search Engine (VarSome, https://varsome.com/) and according to pathogenicity scores adopted by the American College of Medical Genetics and Genomics (ACMG)/Association for Molecular Pathology (AMP) as criteria for variant pathogenicity assessment. Detected variants were defined as novel because of their absence in publicly available databases: The Human Gene Mutation Database (HGMD^®^, www.hgmd.cf.ac.uk), the Leiden Open Variation Database (LOVD v3.0, https://databases.lovd.nl) and the ClinVar (https://www.ncbi.nlm.nih.gov/clinvar), as well as in the literature. The used variation nomenclature followed guidelines indicated by the Human Genome Variation Society (HGVS), based on nucleotide and protein numberings of the Locus Reference Genomic (LRG) sequence format adopted (NCBI refseq NM_001105242, ENSEMBL refseq ENST00000268314.9).

## Outcome and Follow-Up

The 6-months post-surgical evaluation showed biochemical improvement of hormonal secretion parameters, as ACTH levels became detectable and cortisol suppression after 1 mg DST improved, however without reaching normal values (5.8 ug/dl, NR < 1.8 ug/dl). Interestingly, serum IgG levels normalized at 24-months follow-up, while slight low serum levels IgM persisted as well as the increased number of CD3+ T-cells (Table 1). To exclude the possible presence of meningioma, patient underwent brain contrast-enhanced MRI, with the incidental finding of partial empty-sella.

At the last follow-up, after 33 months from adrenalectomy, no further changes in immunological findings were observed. Interestingly, since adrenalectomy the patient did not develop recurrent HSV infection or other viral infections.

## Discussion

We hereby describe the first case, to our knowledge, of a patient affected by PBMAH affected by novel *ARMC5* genetic variants, with slight hypogammaglobulinemia and monthly relapsing human herpes simplex viral infections. This case suggests a possible link between certain ARMC5 mutations and immune system impairment also in humans, as previously described in animal models (Hu et al., 2017).

It is known that CS patients show a variable degree of increased susceptibility to infections, being one of the crucial factors linked to increased morbidity and mortality in these patients. This higher susceptibility is supposed to be based on an increase in white blood cell and neutrophil counts with a decreased lymphocyte count. Glucocorticoids are key regulators of the immune system activation by multiple mechanisms affecting both humoral and cell-mediated responses, resulting in a biphasic dose–response curve, with immunostimulatory effects at low concentrations and suppressive effects at high concentrations. To date, no data are available about the immune system function in patients with subclinical hypercortisolism and the knowledge of the effect of endogenous GCs on Igs concentration is scarce and limited to specific clinical contexts (Berger et al., 1978; Hamilos et al., 1992). Recently, Hu and colleagues added new insight in the role of *ARMC5* gene discovering that *ARMC5* KO mice had compromised T-cell proliferation and differentiation into Th1 and Th17 cells, increased T-cell apoptosis, reduced severity of experimental autoimmune encephalitis, and defective immune responses to lymphocytic choriomeningitis virus infection (Hu et al., 2017). These mice also developed an adrenal gland hyperplasia in old age. No differences were found as for humoral immune responses, and KO mice serum IgG levels were comparable to those of wild-type (WT) controls.

The patient described in our report presented normal neutrophils, an increased T-lymphocytes count with normal CD4+/CD8+ ratio, slightly low serum IgG levels and recurrent labial HSV infections. Both the HSV infections and the slight hypogammaglobulinemia could be considered as manifestations of impaired T-cell function. It is known that patients with overt hypercortisolism are at increased risk of infections linked to alteration of both humoral and cell-mediated immune response (Hasenmajer et al., 2020) however, the patient here described is affected by subclinical hypercortisolism in the absence of any other clinical feature related to cortisol excess. Despite the absence of any other “typical” clinical feature of cortisol excess, our patient underwent unilateral adrenalectomy, which is nowadays considered as the optimal approach to PBMAH, due to lower complication rates as compared to bilateral adrenalectomy, with established improvement of cortisol-related comorbidities. Removal of the largest adrenal was performed, in accordance to available literature (Vassiliadi and Tsagarakis, 2019). Nevertheless, we can not exclude that a chronic and subtle exposure to increased glucocorticoid levels might have led to a specific impairment of thymic epithelial cell function and consequently to an increase of dysfunctional T-cell population mediated by the ARMC5 mutation in this patient (Savino et al., 2016). In this clinical scenario, the reduction of IgG levels and the clinical appraisal of HSV relapsing infections might be related to the progression of hypercortisolism and explain the resolution of both the conditions observed after treatment. In this view, we could argue that the genetic derangement determined by germline *ARMC5* mutations could remain clinically silent unless a concomitant condition is able to further impair immune system function. Further investigations are needed to confirm the possible effects of *ARMC5* mutations on T-cell activity in humans.

## Data Availability

The datasets presented in this article are not readily available due to ethical and privacy restrictions. Requests to access the datasets should be directed to the corresponding author.
